# Colour Doppler analysis of ophthalmic vessels in the diagnosis of carotic artery and retinal vein occlusion, diabetic retinopathy and glaucoma: systematic review of test accuracy studies

**DOI:** 10.1186/s12886-016-0384-0

**Published:** 2016-12-07

**Authors:** Mario Bittner, Livia Faes, Sophie C. Boehni, Lucas M. Bachmann, Reinier O. Schlingemann, Martin K. Schmid

**Affiliations:** 1 Eye Clinic, Cantonal Hospital of Lucerne, Spitalstrasse, CH-6001 Lucerne, Switzerland; 2 medignition Inc, Verena Conzett-Strasse 9, CH-8004 Zurich, Switzerland; 3 Department of Ophthalmology, Academic Medical Center, PO Box 22660, 1100 DD Amsterdam, The Netherlands

## Abstract

**Background:**

Colour Doppler analysis of ophthalmic vessels has been proposed as a promising tool in the diagnosis of various eye diseases, but the available diagnostic evidence has not yet been assessed systematically. We performed a comprehensive systematic review of the literature on the diagnostic properties of Colour Doppler imaging (CDI) assessing ophthalmic vessels and provide an inventory of the available evidence.

**Methods:**

Eligible papers were searched electronically in (Pre) Medline, Embase and Scopus, and via cross-checking of reference lists. The minimum requirement to be included was the availability of original data and the possibility to construct a two-by-two table. Study selection, critical appraisal using the QUADAS II instrument and extraction of salient study characteristics was made in duplicate. Sensitivity and specificity was computed for each study.

**Results:**

We included 11 studies (15 two-by-two tables) of moderate methodological quality enrolling 820 participants (range 30 to 118). In 44.4% participants were female (range 37–59% in specific subgroups). CDI was assessed for internal carotid stenosis, diabetic retinopathy, glaucoma, and branch or central retinal vein occlusion diagnosis. There was insufficient data to pool the results for specific illnesses. For the assessments of ophthalmic arteries, mean sensitivity was 0.69 (range 0.27–0.96) with a corresponding mean specificity of 0.83 (range 0.70–0.96). Mean sensitivity of the central retinal artery assessments was 0.58 (range 0.31–0.84) and the corresponding mean specificity was 0.82 (range 0.63–0.94).

**Conclusions:**

Robust assessments of the diagnostic value of colour Doppler analysis remain uncommon, limiting the possibilities to extrapolate its true potential for clinical practice.

PROSPERO 2014:CRD42014014027.

## Background

Colour Doppler analysis of ophthalmic vessels has been proposed as a promising tool in the diagnosis of various eye diseases [1]. Colour Doppler imaging (CDI) enables ophthalmologists to examine ocular blood flow, even in presence of dense ocular opacities preventing a direct view to the posterior eye segment [2]. Compared to fluorescein angiography (FA), CDI allows for assessing the ocular blood flow in a non-invasive manner. Numerous studies have examined changes in ocular blood flow velocities in various diseases affecting the eye such as carotid artery and retinal vein occlusion, diabetic retinopathy (DRP) and glaucoma, and have revealed significant differences compared to healthy subjects even in early stages of disease (reviewed in [3, 4]). As an example, Evans and colleagues [5] demonstrated that diabetic patients with minimal or no retinopathy demonstrate irregular flow levels in the major vessels feeding the eye.

However, despite several potential benefits, CDI has not accomplished to make the leap into daily ophthalmic routine. Inter-observer variability issues could be a possible explanation [6]. Although updated assessment protocols have enhanced CDI inter-observer variability performance, [7] this remains a limiting factor for implementation in daily clinical use. Another problem relates to the fact that evidence on diagnostic accuracy is scattered among the various clinical fields and presumably not easy to access. For this reason we performed a comprehensive systematic review of the diagnostic literature on ophthalmic Doppler analysis, with special reference to the diagnostic merits of the technique, and provide an inventory of the available evidence.

## Methods

This review has been conducted according to the PRISMA statement recommendations. The review protocol has been registered on Prospero [8] (PROSPERO 2014:CRD42014014027).

### Literature search

An information specialist performed electronic searches without any language restrictions on (pre)MEDLINE (PubMed interface), Scopus and Embase (Ovid-Interface). The full search algorithms for the three databases are provided in the Appendix. Searches were performed on the 11^th^ November 2014 and updated on the 4^th^ of May 2016.

### Eligibility criteria

We excluded studies assessing only healthy subjects, animal studies and studies without primary focus on eye diseases. We included case-control and cohort studies. The minimum requirement was the availability of original data and the possibility to construct a two-by-two table. We accepted the following reference tests classifying disease presence: carotid angiography, carotid ultrasound, fundoscopy, Humphrey visual field analyser, fluorescein angiography and Heidelberg retina optical coherence tomography.

### Study selection, data extraction and quality assessment

Based on the QUADAS II criteria all eligible papers were judged for their quality using four suggested domains (patient selection, index test, reference standard and flow and timing) [9]. The instrument assesses the risk of bias of each domain. Moreover, the analysis of patient selection, the index test and the reference test examine the extent of applicability. The tool is broadly applied to standardise the rating of bias and to weigh applicability of diagnostic accuracy studies. We followed the recommendations of Whiting and colleagues [9] and refrained from rating or ranking of studies. Two reviewers independently read and assessed papers and extracted data using a standardized data abstraction form. Discrepancies were resolved by consensus between the two reviewers.

### Statistical analysis

For each study, we constructed a two-by-two contingency table consisting of true-positive (TP), false-positive (FP), false-negative (FN), and true-negative (TN) results. For the analysis, we called a result a true positive if the CDI finding was concordant and in agreement with the reference standard findings. We calculated sensitivity as TP/(TP + FN) and specificity as TN/(FP + TN).

### Meta- analysis

Formal pooling was only planned if at least four studies reported accuracy parameters for the diagnosis of a specific illness. However, in an exploratory meta-analysis of sensitivity and specificity using a multilevel mixed-effects logistic regression model (metandi command in Stata), we calculated diagnostic summary estimates mainly of the resistance index measured either in the ophthalmic or the central retinal artery irrespective of the underlying condition. This was done to gain an overall impression about the discriminatory potential of the technology rather than to inform clinical practice.

All analyses were done using the Stata 14.1 statistical software package (StataCorp. 2015. Stata Statistical Software: Release 14. College Station, TX: StataCorp LP.).

## Results

### Study selection

**Fig. 1:**
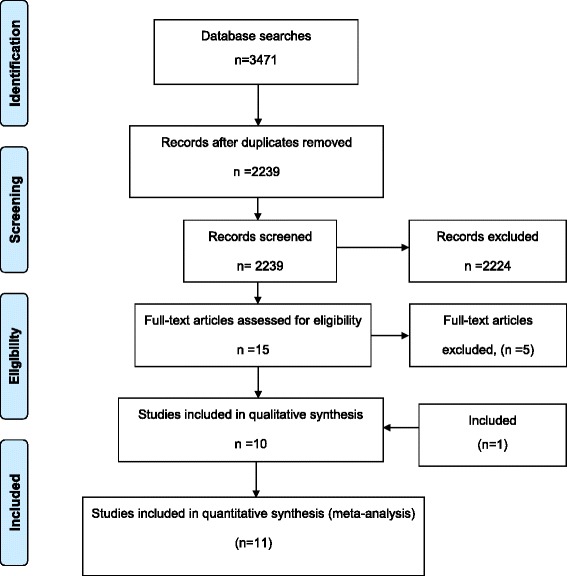
Study selection process (PRISMA Statement flowchart)

Electronic searches retrieved 3471 records. After excluding duplicates, 2239 records remained. Screening of titles and abstracts excluded 2224 studies as they did not investigate the diagnostic accuracy of tests, investigated other conditions, or contained no primary data. Full texts of fifteen articles were read. Five of these studies did not meet the inclusion criteria and were excluded. The ten remaining articles were completed by addition of another study found via the screening of reference lists. In total, thus, 11 studies were included in this systematic review. Figure 1 shows the study selection process.

### Quality of included studies

We assessed the quality of the included studies using the QUADAS II tool considering four criteria (patient selection, index test, reference standard and flow and timing). The reference standard was appropriate in all selected trials, whereas flow and timing of the studies was inconsistent, mostly due to long periods of disease onset to performance of index- and reference tests. Most studies scored low for the items patient selection and rating of the index test. In case of patient selection this was partly due to a lack of age and sex matched study groups and partly due to not describing exclusion criteria. Low ratings regarding the index test were related to cut-off points that were not pre-specified and to the fact that not all investigators performed the index test being unaware of the diagnosis. Table 1 outlines the results of the quality assessment according to QUADAS II criteria.

**Table 1:** Study characteristics, patient population

Name, year	Diagnosis in study population	Design	Consecutive	n(eyes)	Mean age (years)	Female population	Patient selection	Index test	Reference standard	Flow and timing
Han-Hwa Hu, 1993 [10]	CIS <50%: *n* = 84; CIS 50–74%: *n* = 23; CIS 75–99%: *n* = 17; CIS 100%: *n* = 8	Cross sectional study	n.r.	132	63	11%	2	2	1	1
Paivansalo,1999 [11]	both side CIS <80%: *n* = 46 ; at least one side CIS >80%:*n* = 48	Cross sectional study	n.r.	94	63.1	38%	2	2	1	1
Arai, 1998 [12]	DM without DRP: *n* = 27, DRP: *n* = 25	Cross sectional study	n.r.	52	DM without DRP: 50.0, DRP: 56.1	45%	1	2	1	3
Basturk, 2012 [13]	DM II without DRP: *n* = 40, DRP: *n* = 51, healthy subjects: *n* = 27	Cross sectional study	yes	118	DRP: 55.88, DM without DRP: 53.25, healthy subjects 53.59	DM without DRP: 45%, DRP: 49%; healthy controls: 48%	1	2	1	3
Jimenez-Aragon, 2013 [14]	Early glaucoma no-progression group: *n* = 59; early glaucoma progression group: *n* = 12	Longitudinal and prospective study	yes	71	No progression group 54.03, Progression group 55.75	No-Progression group: 59%; Progression group: 75%	1	1	1	3
Arsène, 2002 [18]	CRVO-ischaemic: *n* = 18; CRVO-non-ischaemic: *n* = 51; BRVO (ischaemic and non-ischaemic):*n* = 33	Longitudinal and prospective study	n.r.	102	61	38%	2	1	1	2
Tranquart, 1999 [19]	CRVO-ischemic: *n* = 18; CRVO: non-ischaemic: *n* = 51	Cross sectional study	n.r.	69	61 (based on 102 subjects)	38% (based on 102 subjects)	2	1	1	3
Plange, 1999 [17]	NTG: *n* = 62; healthy controls: *n* = 40	Cross-sectional study	yes	102	NTG: 57, healthy subjects: 58	37%	1	1	1	1
Martinez, 2005 [15]	POAG progression group: *n* = 23; POAG no-progression group: *n* = 26	Prospective cohort study	yes	49	63.7	53%	1	3	1	1
Suprasanna, 2014 [16]	POAG-progression group: *n* = 25; POAG no-progression-group: *n* = 53	Case control study	n.r.	78	62.6	41%	2	2	1	1
Williamson, 1994 [6]	CRVO without iris neovascularization: *n* = 22, CRVO with iris neovascularization: *n* = 8	Prospective cohort study	n.r.	30	67.2 (based on 80 patients)	49% (based on 80 subjects)	1	2	1	2

*n.r.* not reported, *CIS* carotis interna stenosis, *DM* diabetes mellitus, *DRP* diabetic retinopathy, *CRVO* central retinal vein occlusion, *NTG* normal tension glaucoma, *POAG* primary open angle glaucoma, 1 = low risk, 2 = high risk, 3 = unclear risk

### Patients’ characteristics, design features

The 11 selected studies enrolled 820 patients. Among the studies which reported this, 44.4% of participants were women ranging from 11 to 75%. Study size ranged between 30 and 118 subjects. Four studies included patients consecutively. The most commonly described co-morbidities (other than the assessed illness) were hypertension and diabetes. Patients’ characteristics are summarized in Table 1.

### Index and reference tests

Two studies assessed the possibility of a >75% or >80% internal carotid stenosis (ICS) diagnosis [10, 11]. Another two trials assessed the diagnosis of DRP. Whereas Arai et al. [12] examined the diagnosis of DRP among patients suffering from non-insulin-dependent diabetes, Basturk and colleagues [13] investigated the diagnosis of DRP in patients suffering from diabetes type II with microalbuminuria. Prediction of glaucoma progression was investigated in three studies two using the Humphrey field analyser and one the Heidelberg retina tomography as the reference test [14–16]. One study aimed to distinguish normal tension glaucoma from healthy eyes [17]. In another two studies, fluorescein angiography was set as the reference test assessing diagnosis of branch or central retinal vein occlusion (BRVO, CRVO) and diagnosis of ischemic CRVO respectively [18, 19]. Arséne et al. [18] aimed to distinguish eyes with CRVO from eyes with BRVO. Gonioscopy and slit lamp examination were set as reference tests in one study predicting iris vascularization 1 year after CRVO onset [6]. Table 2 shows the applied index and reference tests of each study.

**Table 2:** Summary of index and reference tests applied in included studies

Name, year	Index test	Reference tests								
	CDI	CA	cCDI	FS	HRT	GAT	SLE	FLA	HFA	GS
Han-Hwa Hu, 1993 [10]	x	x	x							
Paivansalo, 1999 [11]	x		x							
Arai, 1998 [12]	x			x						
Basturk, 2012 [13]	x			x						
Jimenez-Aragon, 2013 [14]	x				x					
Arsène, 2002 [18]	x					x	x	x		
Tranquart, 1999 [19]	x					x	x	x		
Plange, 1999 [17]	x			x					x	
Martinez, 2005 [15]	x								x	
Suprasanna, 2014 [16]	x								x	
Williamson, 1994 [6]	x						x			x

*CDI* colour doppler imaging, *CA* carotid angiography, *cCDI* carotid colour doppler imaging, *FS* fundoscopy, *HRT* Heidelberg retina tomograph, *GAT* Goldmann applanations tonometry, *SLE* slit lamp examination, *FLA* flourescein angiography, *HFA* Humphrey field analyzer, *GS* gonioscopy

### Index test parameters assessed

Of the four studies assessing glaucoma, two used the resistive index (RI) to evaluate test accuracy of glaucoma progression [15, 16]. Another study used diastolic velocity to distinguish between normal tension glaucoma (NTG) and healthy eyes [6] and one study used a logistic discrimination function to predict the progression of glaucomatous damage [14]. Of the two studies assessing ICS, one used peak flow velocity [10] and one the RI [11]. Both studies examining the diagnostic value of CDI in DRP used RI as investigational parameter [12, 13]. Two studies assessing ischemia and neovascularization after CRVO, used the parameter “minimum velocity” [6, 19]. One study assessed the possibility to distinguish between CRVO and BRVO used the RI [18].

### Test performance characteristics

Among studies assessing the ophthalmic artery (OA) flow, mean sensitivity was 0.69 (range 0.27–0.96) with a corresponding mean specificity of 0.83 (range 0.70–0.96), whereas for central retinal artery (CRA) flow measurements, mean sensitivity was 0.58 (range 0.31–0.84) with a corresponding specificity of 0.82 (range 0.63–0.94). The range of the number of eyes assessed in studies examining OA was 49 to 132, and 52 to 132 in studies assessing CRA. Assessments of the central retinal vein (CRV) reported sensitivities ranging from 0.67 to 0.75 and specificities of 0.65 to 0.86 in 30 and 69 eyes respectively. All test performance characteristics are outlined in Table 3.

**Table 3:** Test performance characteristics of included studies

Name, year	Vessel	Pathology	TP	FP	FN	TN	Sensitivity	Specificity	negative LR (95% CI)	positive LR (95% CI)
Han-Hwa Hu, 1993 [10]	oa^a^	0	21	20	4	87	85.70%	80.90%	0.197 (0.080 to 0.485)	4.49 (2.92 to 6.91)
Jimenez-Aragon, 2013 [14]	oa	2	7	6	5	53	54.60%	90.00%	0.464 (0.236 to 0.911)	5.74 (2.34 to 14.06)
Martinez, 2005 [15]	oa	2	19	2	4	24	82.60%	92.30%	0.188 (0.077 to 0.462)	10.74 (2.80 to 41.21)
Suprasanna, 2014 [16]	oa	2	24	9	1	44	96.00%	83.00%	0.048 (0.007 to 0.330)	5.65 (3.10 to 10.31)
Basturk, 2012 [13]	oa	1	40	20	11	47	78.40%	70.00%	0.307 (0.178 to 0.531)	2.63 (1.77 to 3.90)
Paivansalo, 1999 [11]	oa	0	13	2	35	44	27.00%	96.00%	0.762 (0.635 to 0.915)	6.23 (1.49 to 26.10)
Arai, 1998 [12]	oa	1	14	8	11	19	56.00%	72.00%	0.625 (0.377 to 1.037)	1.89 (0.96 to 3.72)
Jimenez-Aragon, 2013 [14]	cra^a^	2	6	4	6	55	50.00%	94.00%	0.536 (0.303 to 0.948)	7.38 (2.45 to 22.21)
Arai, 1998 [12]	cra	1	16	10	9	17	64.00%	63.00%	0.572 (0.315 to 1.039)	1.73 (0.97 to 3.06)
Han-Hwa Hu, 1993 [10]	cra	0	21	11	4	96	84.00%	89.60%	0.178 (0.072 to 0.439)	8.17 (4.55 to 14.67)
Paivansalo, 1999 [11]	cra	0	15	6	33	40	31.00%	88.00%	0.791 (0.634 to 0.986)	2.4 (1.02 to 5.64)
Arsène, 2002 [18]	cra	3	48	11	21	22	70.00%	66.00%	0.457 (0.297 to 0.702)	2.09 (1.26 to 3.47)
Plange, 1999 [17]	cra	2	30	4	32	36	48.40%	90.00%	0.573 (0.441 to 0.745)	4.84 (1.84 to 12.70)
Tranquart, 1999 [19]	crv^a^	3	12	18	6	33	67.00%	65.00%	0.515 (0.260 to 1.021)	1.89 (1.15 to 3.10)
Williamson, 1994 [6]	crv	3	6	3	2	19	75.00%	86.00%	0.289 (0.086 to 0.972)	5.5 (1.79 to 16.94)

*TP* true positives, *FP* false positives, *FN* false negatives, *TN* true negatives, *LR* likelihood ratio, *CI* confidence interval

^a^ *oa* ophthalmic artery, *cra* central retinal artery, *crv* central retinal vein

Pathology: 0 = central carotic stenosis; 1 = diabetes; 2 = glaucoma; 3 = central retinal vein occlusion

### Results from exploratory meta-analysis

**Fig. 2:**
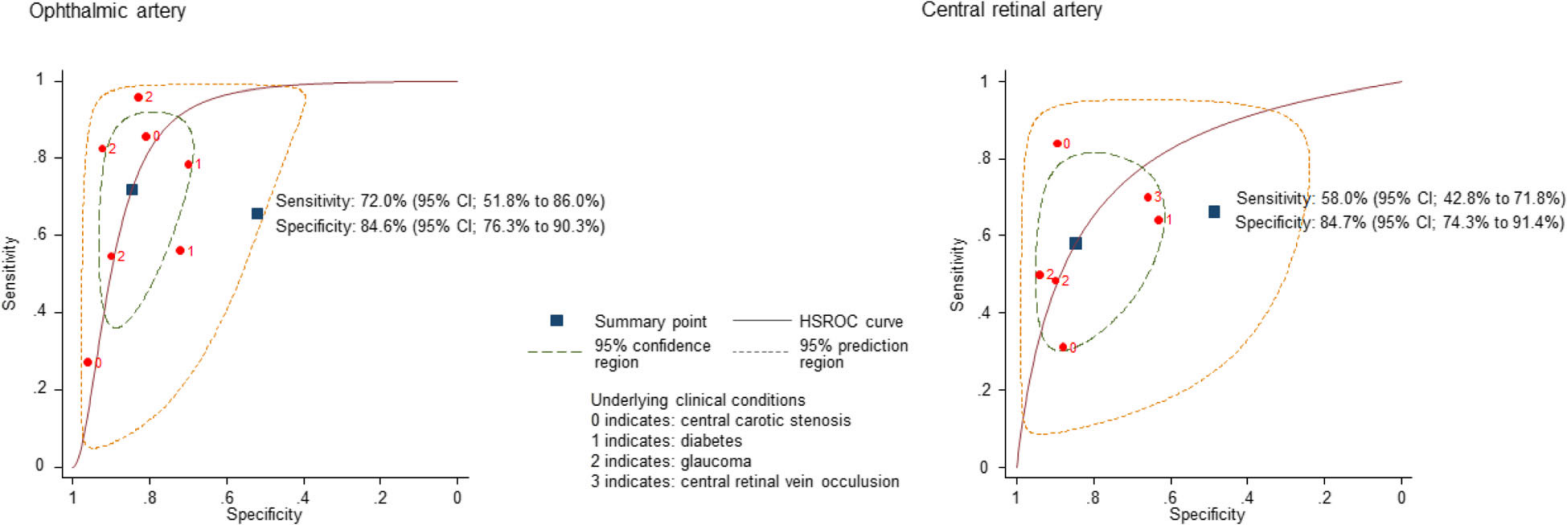
Hierarchical summary ROC curve of studies assessing Ophthalmic (*left*) and Central retinal (*right*) artery

Formal pooling of results for specific illnesses was impossible due to the limited number of studies. In the exploratory meta-analysis of studies investigating the OA across different illnesses, the pooled estimated sensitivity was 0.72 (95% CI; 0.52 to 0.86) with a corresponding specificity of 0.85 (95% CI; 0.76 to 0.90). The analysis of the CRA revealed a lower sensitivity of 0.58 (95% CI; 0.43 to 0.72) and a comparable specificity of 0.85 (95% CI; 0.74 to 0.91) (see Fig. 2).

## Discussion

### Main findings

The results of a small number of studies with only few patients suggest that the diagnostic value of colour Doppler analysis in ophthalmology is limited. Diagnostic performance varies considerably between studies illnesses with slightly better results when assessing the OA. Even though alterations of ocular blood flow could be used as a valuable tool in diagnosing ocular pathologies their acquisition remains a technical challenge and a no gold standard for assessment exists due to inherent limitations of the available techniques [4, 20]. Another problem in daily business is the lack of standardized methods in CDI assessment as Stalmans et al. already indicated [21]. Despite the vast amount of studies assessing ocular diseases, only a minority quantifies the CDI’s accuracy, impeding judgments regarding its value in the diagnostic work-up of ocular pathologies.

### Results in context of existing literature

To our knowledge, this is the first comprehensive assessment of studies examining the diagnostic value of CDI in ocular diseases. We are aware of one meta-analysis of studies dealing with hemodynamic changes in ophthalmic artery and central retinal artery using CDI in Chinese patients with glaucoma [22]. However, this meta-analysis does not assess the diagnostic value using test accuracy data of CDI in glaucomatous patients [23]. In contrast to our findings, Dimitrova et al. [2] published a review stating CDI as a valuable tool in a wide range of ophthalmologic diseases for research and as a potentially useful diagnostic tool in the clinical setting. Although CDI appears to be a useful tool in the research of ocular blood flow changes, especially in glaucoma, diabetic retinopathy and age related macular degeneration [24–29], implementation in daily use as a diagnostic tool concerning the pathologies assessed in our study cannot be recommended yet.

### Strengths and limitations

Our study applied up-to-date systematic review methodology, allowing a comprehensive description of the existing diagnostic literature on CDI test accuracy in ophthalmology. Due to the limited number of studies and the small number of participants in the included studies more upstream and formally sound (statistical) analyses were not feasible. We performed exploratory analyses to give an overview of the diagnostic performance of the CDI. However, the analysis has major limitations due to the mix of studies investigating different conditions and some methodological weaknesses of the underlying studies. Also, no analysis of the impact of different CDI systems on diagnostic performance could be performed due to the paucity of data. Hence we could not go beyond a mere description of the evidence.

### Implications for further research

For successful implementation in clinical care, standards of CDI assessment need to be introduced to form the basis of a consistent assessment tool and to decrease the potential inter-rater reliability issues. There are promising results of altered ocular blood flow in common ocular diseases like diabetic retinopathy, age related macular disease and several forms of glaucoma [24, 27–31] and growing evidence showing that ocular blood flow alterations are associated with the development and progression of ophthalmologic diseases confirm the notion that abnormalities in vessel function exist before the development of structural changes occur. Considering this exciting point of view it seems promising to focus research of preclinical changes of ocular blood flow and prediction of progression of respective diseases. In order to fully understand underlying pathophysiological mechanisms and to offer timely treatments further studies are needed to fill in the current lack of knowledge.

### Implications for practice

For the pathologies assessed in our analysis, implementation of CDI as a diagnostic tool in clinical practice does not seem helpful. Current available diagnostic tools like slit lamp examination, fluorescence angiography, visual field analysers and scanning laser polarimetry seem to be superior and more practicable compared with CDI. In case of glaucoma, the diagnosis relies on the assessment of intraocular changes occurring in the visual field, the optic nerve head, and the retinal nerve fibre layer. Perimetry is the current gold standard of progression as it enables the detection of small glaucomatous changes to the visual field. However, perimetry is limited by low diagnostic sensitivity in early stages of the disease [32] and there is a need for assessment techniques capable of detecting preclinical stages of the disease. In case of retinal vein occlusion fundoscopy and fluorescence angiography are satisfying diagnostic tools. However, predicting ischemic areas using alterations of ocular blood flow could be a valuable non-invasive tool. Regarding diabetic retinopathy, again, slit lamp examination and fluorescence angiography are valuable diagnostic tools superior to CDI. However, early detection of altered blood flow could improve implementation of therapy in early stages of the disease.

## Conclusions

The possible role of colour Doppler analysis in routine ophthalmologic care remains unclear. The relative lack of robust assessments of the diagnostic value of colour Doppler analysis, limits the possibilities to extrapolate its true potential for clinical practice.

## Abbreviations

BRVO: Branch vein occlusion
CDI: Colour Doppler imaging
CRVO: Central retinal vein occlusion
DRP: Diabetic retinopathy
FA: Fluorescein angiography
FN: False-negative
FP: False-positive
HSROC: Hierarchical summary receiver operating characteristics curve
ICS: Internal carotid stenosis
NTG: Normal tension glaucoma
RI: Resistive index
TN: True-negative
TP: True-positive
